# Fowl Adenovirus Serotype 4 SD0828 Infections Causes High Mortality Rate and Cytokine Levels in Specific Pathogen-Free Chickens Compared to Ducks

**DOI:** 10.3389/fimmu.2018.00049

**Published:** 2018-01-25

**Authors:** Rong Li, Gen Li, Jing Lin, Shaojie Han, Xiaolan Hou, Hongyu Weng, Mengjiao Guo, Zhong Lu, Ning Li, Yingli Shang, Tongjie Chai, Liangmeng Wei

**Affiliations:** ^1^College of Animal Science and Veterinary Medicine, Sino-German Cooperative Research Centre for Zoonosis of Animal Origin of Shandong Province, Shandong Provincial Key Laboratory of Animal Biotechnology and Disease Control and Prevention, Shandong Provincial Engineering Technology Research Center of Animal Disease Control and Prevention, Shandong Agricultural University, Tai’an, China; ^2^Collaborative Innovation Center for the Origin and Control of Emerging Infectious Diseases, Taishan Medical University, Tai’an, China

**Keywords:** fowl adenovirus serotype 4, hydropericardium-hepatitis syndrome, specific pathogen-free chicken, specific pathogen-free duck, pathogenicity, innate immune response, antiviral ability

## Abstract

Hydropericardium syndrome and inclusion body hepatitis, together called hydropericardium-hepatitis syndrome, are acute infectious diseases found in chickens. These diseases are caused primarily by fowl adenovirus serotype 4 (FAdV-4) strains. In this study, we isolated a FAdV-4 strain (SD0828) from clinically diseased chickens and phylogenetically analyzed the L1 loops of the hexon protein sequences in 3-week-old specific pathogen-free chickens and ducks infected intramuscularly and orally, determining differences in the pathogenicity by observing clinical signs and gross and histological lesions. We also detected the viral load in tissue samples. Postinfection necropsy showed that all chickens but no ducks exhibited typical necropsy lesions. Additionally, all chickens infected intramuscularly died within 2 days postinfection (dpi), and all those infected orally died within 5 dpi, whereas no infected ducks died before 28 dpi. Quantitative real-time polymerase chain reaction analysis was used to determine the viral load in the tissues of hearts, livers, spleens, lungs, and kidneys and in cloacal cotton swabs from infected chickens and ducks at 1, 2, 3, 5, 7, 14, 21, and 28 dpi. The greatest number of viral DNA copies was found in the livers of infected chickens, yet no virus was found in any samples from infected ducks. In addition, the viral load increased over time in both chicken and duck embryo fibroblasts (CEFs and DEFs, respectively); in the former, replication speed was significantly greater than in the latter. Innate immune responses were also studied, both *in vivo* and *in vitro*. In CEFs, DEFs, and chickens infected intramuscularly, but not in infected ducks, mRNA expression levels of proinflammatory cytokines (interleukin-6 and -8) and interferon-stimulated genes (Mx and OAS) were significantly upregulated. Although some cytokines showed significant upregulation in the oral chickens, most did not change significantly. Finally, the duck retinoic acid-inducible gene I and its caspase activation and recruitment domain both had significant antiviral functions in CEFs, particularly after 24 h postinfection. Taken together, this research provides new insights into the interactions between FAdV-4 and the innate immune systems of studied hosts (chickens and ducks).

## Introduction

Hydropericardium-hepatitis syndrome (HHS) is caused by fowl adenovirus serotype 4 (FAdV-4). It was first seen in the Pakistan Angara Goth in 1987; therefore, it has also been called “Angara Disease” (1, 2). This serotype is non-enveloped with double-stranded DNA, belonging to the adenoviruses (AdVs); the family of AdVs is currently grouped into five genera: (a) mastadenovirus, (b) aviadenovirus, (c) atadenovirus, (d) siadenovirus, and (e) ichtadenovirus (3). All FAdVs belong to the Aviadenovirus genus, and they are divided into five species (FAdV-A, -B, etc.), and these are further subdivided into 12 serotypes based on molecular structure and a serum cross-neutralization test (4, 5). These serotypes belong to the five species as follows: (a) FAdV-A includes FAdV-1; (b) FAdV-B, FAdV-5; (c) FAdV-C, FAdV-4 and -10; (d) FAdV-D, FAdV-2, -3, -9, and -11; and (e) FAdV-E, FAdV-6, -7, -8a, and -8b (6). The L1 loop of the hexon is the most abundant viral surface protein, and it contains major antigenic determinants (7). Phylogenetic analysis of its sequences is both cost-effective and a complementary identification method for AdV genotyping (8, 9).

In recent years, hydropericardium syndrome has been reported in Iraq, Kuwait, India, Mexico, Ecuador, Peru, Chile, Korea, Japan, and South and Central Americas (10, 11). Since 2014, it has posed a serious threat to the broiler industry in most parts of China (7). It is caused primarily by FAdV-4 (12, 13), and it affects 3–6-week-old boiler chickens (14). It is accompanied by hydropericardium and results in mortality rates of 30–70% (15). Its clinical manifestation is seen as depression, lethargy, ruffling of neck feathers, and decreased feed intake (11). Necropsy reveals swollen and pale-colored liver and swollen and bleeding kidneys. The typical necropsy lesion consists of very light yellow jelly-like or watery pericardial extravasations (13, 16). In addition, the main finding upon pathological examination is a large number of intranuclear inclusion bodies in the liver (11, 17).

The first line of defense against microbial infection is a range of pattern recognition receptors (PRRs), consisting primarily of four families: Toll-like receptors (TLRs), nucleotide-binding oligomerization-domain-like receptors (NLRs), retinoic acid-inducible gene I (RIG-I)-like receptors, and C-type lectin families (18, 19). After viral infection, some PRRs, such as TLR3, TLR7, and RIG-I, are activated (20, 21), and once activated, stimulate downstream signaling cascades and produce an immune response (22). As the primary member of RIG-I-like receptors, RIG-I possesses caspase activation and recruitment domains (CARDs) at the N-terminal (23), allowing it to specifically recognize various types of viral nucleotides in the cytoplasm (24). Previous studies show a natural resistance to influenza viruses in ducks, linked to RIG-I, but chickens are missing this gene, providing a plausible explanation for their increased susceptibility to influenza compared to ducks (25).

Numerous studies show that duck AdV can cause diseases in ducks. For example, it can induce decreased egg production and egg quality (26). A specific form DAdV-3 (CH-GD-12-2014) can cause pathologically different hydropericardium and liver hemorrhaging in infected specific pathogen-free (SPF) ducks (27). Combined with the acute and fatal events caused by FAdV-4 in chickens, it is interesting to learn whether ducks have the same or similar symptoms after FAdV-4 infection. Chen’s study suggests that FAdV-4 also causes hydropericardium in ducks, resulting in death after intracerebral injection (28). We comparatively studied the differences in pathogenicity between SPF chickens and SPF ducks and examined the various antiviral innate immune responses in these two fowl. We further investigated whether RIG-I plays an important role after FAdv-4 infection and examined the antiviral functions of duck RIG-I and RIG-I-CARD in FAdV-4-infected chicken embryo fibroblasts (CEFs).

## Materials and Methods

### Viruses, Cells, and Animals

In this study, a specific FAdV-4 strain, SD0828, was isolated from chicken livers clinically diseased with HHS by the Environmental Microbiology Laboratory at Shandong Agricultural University. The virus was plaque-purified, and SD0828 was propagated and titrated in continuous CEFs as described elsewhere (29). Nine-day-old chicken embryos were used to make CEFs, and the viral titer for infection was determined using the median tissue culture infective dose (TCID_50_) method (30).

Duck embryo fibroblasts (DEFs), derived from 12-day-old SPF duck embryos, and CEFs were cultured in Dulbecco’s modified Eagle medium (Gibco, Grand Island, NY, USA) that included 10% fetal bovine serum (Transgen, Beijing, China). They were incubated at 37°C in 5% (v/v) CO_2_.

To evaluate FAdV-4-SD0828 pathogenicity in chickens and ducks, 91-day-old SPF White Leghorn chickens (Jinan) and an equal number of 1-day-old SPF Shao Xing ducks (Harbin) were used. All animals were housed in isolation and handled in strict accordance with the guidelines of the Shandong Agricultural University Animal Care and Use Committee. The approval number for this study is SDAU-2016-001.

### Molecular Cloning and Phylogenetic Analysis

The viral DNA load of SD0828 in clinically diseased chicken livers was extracted using viral DNA kits (Omega, CA, USA) following manufacturer’s instructions. Specific polymerase chain reaction (PCR) primers for SD0828-F1 and -R1 were designed so that the entire viral hexon gene sequence could be identified (Table 1) and then sequenced by Qingdao Tsingke Zixi Biological Technology. For phylogenetic analysis, the L1 loop of the hexon protein (base pairs 145–1,041) of the CELO strain (GenBank number AF339914) was selected for referencing (31). All FAdV strains used in this study were found on the National Center for Biotechnology’s website and referenced to previous studies (8, 32). The amino acid sequence GenBank number is shown in Table 2. Megalign software was used to analyze its similarity with and evolution from the L1 loop. A phylogenetic analysis was generated using MEGA 5.1 software, and the tree was constructed using the neighbor-joining method with bootstrapping over 1,000 replicates.

**Table 1:** The primers information.

Primer name	Sequence of oligonucleotide (5′–3′)	Purpose	GenBank number
FAdV-4 F1	CGTCCCGACTACCCTAGAGA	RT-PCR	KU569296.1
FAdV-4 R1	AGGTCCCGCAACTGAGACT		

qc-β-actin F	GAGAAATTGTGCGTGACATCA	qRT-PCR	L08165.1
qc-β-actin R	CCTGAACCTCTCATTGCCA		

qc-TLR3 F	ACAATGGCAGATTGTAGTCACCT	qRT-PCR	NM001011691.3
qc-TLR3 R	GCACAATCCTGGTTTCAGTTTAG		

qc-TLR7 F	TGTGATGTGGAAGCCTTTGA	qRT-PCR	DQ780342
qc-TLR7 R	ATTATCTTTGGGCCCCAGTC		

qc-MDA5 F	TGAAAGCCTTGCAGATGACTTA	qRT-PCR	AB371640.1
qc-MDA5 R	GCTGTTTCAAATCCTCCGTTAC		

qc-IL-1β F	GGTCAACATCGCCACCTACA	qRT-PCR	NM204524.1
qc-IL-1β R	CATACGAGATGGAAACCAGCAA		

qc-IL-6 F	TCTGTTCGCCTTTCAGACCTA	qRT-PCR	AJ309540
qc-IL-6 R	GACCACCTCATCGGGATTTAT		

qc-IL-8 F	GCTCTGTCGCAAGGTAGGAC	qRT-PCR	DQ393272.2
qc-IL-8 R	GCGTCAGCTTCACATCTTGA		

qc-IFN-α F	ATGCCACCTTCTCTCACGAC	qRT-PCR	EU367971
qc-IFN-α R	AGGCGCTGTAATCGTTGTCT		

qc-IFN-β F	TCCTACTGCTCTTGCTTCTGC	qRT-PCR	NM001024836.1
qc-IFN-β R	TGGAAATGGAAAAGTCACGTC		

qc-IFN-γ F	TGAGCCAGATTGTTTCGATG	qRT-PCR	DQ906156/X99774
qc-IFN-γ R	CTTGGCCAGGTCCATGATA		

qc-Mx F	CAGCTCCAGAATGCATCAGA	qRT-PCR	AB244818.1
qc-Mx R	GGCAATTCCAGGAAGATCAA		

qc-OASL F	GAGATGGAGGTCCTGGTGAA	qRT-PCR	NM205041.1
qc-OASL R	CCAGCTCCTTGGTCTCGTAG		

qd-β-actin F	GGTATCGGCAGCAGTCTTA	qRT-PCR	EF667345.1
qd-β-actin R	TTCACAGAGGCGAGTAACTT		

qd-TLR3 F	GAGTTTCACACAGGATGTTTAC	qRT-PCR	JQ910167
qd-TLR3 R	GTGAGATTTGTTCCTTGCAG		

qd-TLR7 F	CCTTTCCCAGAGAGCATTCA	qRT-PCR	AY940195
qd-TLR7 R	TCAAGAAATATCAAGATAATCACATCA		

qd-MDA5 F	GCTACAGAAGATAGAAGTGTCA	qRT-PCR	KJ451070.1
qd-MDA5 R	CAGGATCAGATCTGGTTCAG		

qd-RIG-I F	GCTACCGCCGCTACATCGAG	qRT-PCR	EU363349
qd-RIG-I R	TGCCAGTCCTGTGTAACCTG		

qd-IL-1β F	TCATCTTCTACCGCCTGGAC	qRT-PCR	DQ393268
qd-IL-1β R	GTAGGTGGCGATGTTGACCT		

qd-IL-6 F	TTCGACGAGGAGAAATGCTT	qRT-PCR	AB191038
qd-IL-6 R	CCTTATCGTCGTTGCCAGAT		

qd-IL-8 F	AAGTTCATCCACCCTAAATC	qRT-PCR	DQ393274
qd-IL-8 R	GCATCAGAATTGAGCTGAGC		

qd-IFN-α F	TCCTCCAACACCTCTTCGAC	qRT-PCR	EF053034
qd-IFN-α R	GGGCTGTAGGTGTGGTTCTG		

qd-IFN-β F	AGATGGCTCCCAGCTCTACA	qRT-PCR	KM035791.1
qd-IFN-β R	AGTGGTTGAGCTGGTTGAGG		

qd-IFN-γ F	GCTGATGGCAATCCTGTTTT	qRT-PCR	AJ012254
qd-IFN-γ R	GGATTTTCAAGCCAGTCAGC		

qd-Mx F	TGCTGTCCTTCATGACTTCG	qRT-PCR	GU202170.1
qd-Mx R	GCTTTGCTGAGCCGATTAAC		

qd-OAS F	TCTTCCTCAGCTGCTTCTCC	qRT-PCR	KJ126991.1
qd-OAS R	ACTTCGATGGACTCGCTGTT		

qd-PKR F	AATTCCTTGCCTTTTCATTCAA	qRT-PCR	KR025553.1
qd-PKR R	TTTGTTTTGTGCCATATCTTGG		

*RT-PCR, real-time polymerase chain reaction; qRT-PCR, quantitative real-time polymerase chain reaction; qc, qRT-PCR primer of chickens; qd, qRT-PCR primer of ducks; F, forward primer; R, reverse primer*.

**Table 2:** Sequences information of FAdV reference strains used in present study.

Species	Serotype	Strains	GenBank numbers
FAdV-A	FAdV-1	AdV CELO	U46933.1
		FAdV-1 CELO	AF339914.1
		FAdV-A K181 10	JN181575.1

FAdV-B	FAdV-5	FAdV-B 09-7473-2	FN869988.1
		FAdV-B 160 2011	KC750798.1
		FAdV-B 177 2011	KC750799.1

FAdV-C	FAdV-4	FAdV-4	AJ431719.1
	FAdV-10	FAdV-4 KC	EU177545.1
		FAdV-4 PB-05	EU931691.1
		FAdV-4 PK-01	EU931693.1
		FAdV-4 ATCC VR-829	AF339917.1
		FAdV-4 KR5	AF508951.1
		FAdV-10	U26221.1

FAdV-D	FAdV-2	FAdV-2 685	AF508947.1.
	FAdV-11	FAdV-2 SR48	AF508946.1
		FAdV-11	HQ697595.1
		FAdV-11 C2B	AF508959.2
		FAV-JLPS-141030-B	KU981131.1
		FAV-YTLY-091107-B	KU981123.1

FAdV-E	FAdV-6	FAdV-6 CR119	AF508954.2
	FAdV-7	FAdV-7 ATCC VR-832	AF339922.1
	FAdV-8a	FAdV-8b 764	JN112373.1
	FAdV-8b	FAdV-HNQX-101017-B	KU981154.1
		FAV-JL-130131-B	KU981148.1
		FAV-SDWF-110712-B	KU981142.1

DAdV	DAdV-1	DAdV-1	CAA70809.1
	DAdV-2	DAdV-1	EF093507.1
	DAdV-4	DAdV-2 CH-GD-12-2014	ALF39443.1
		DAdV-2 GR	AIE77222.1
		DAdV-2 GR	YP 009047163.1
		FAdV-4	KX538980.1
		FAdV-4-WFLQ-140704-G	KU981153.1

### *In Vivo* Experimental Procedure

The *in vivo* viral titer was determined to be 10^6.3^ TCID_50_/mL. After three weeks of normal feeding, SPF chickens were randomly divided into three groups of thirty. Group I was infected with 0.1 mL viral stock *via* the intramuscular (IM) route. Group II was infected with the same volume of the same stock *via* the oral route. Controls were inoculated with the same volume of phosphate-buffered saline (PBS) *via* the IM route. At 1, 2, 3, and 5 days postinfection (dpi), three chickens from each group were euthanized, and the hearts, livers, spleens, lungs, kidneys, brains, and glandular stomachs were collected. Samples were fixed with 4% formalin at room temperature for histopathological examination while other parts were stored at −70°C for DNA and RNA extraction. In addition, the peripheral blood mononuclear cells (PBMCs) were also separation from the uninfected and infected chickens with the peripheral blood lymphocyte separation kit (TBDsciences, Tianjin, China) for the RNA extraction. The cloacal cotton swabs of each group were collected at 1, 2, 3, and 5 dpi to test the viral loads. The treatments of SPF ducks were the same as the chickens except the sample collection times were at 1, 2, 3, 5, 7, 14, 21, and 28 dpi.

### *In Vitro* Experimental Procedure for CEFs and DEFs

The *in vitro* viral titer was determined to be 10^3.3^ TCID_50_/mL. Both CEFs and DEFs were seeded in 6-well plates and cultured overnight at 37°C in 5% (v/v) CO_2_. Using 0.5 mL/well viral diluent, CEFs and DEFs were infected, incubated at 37°C for 2 h, then cultured with 2 mL Dulbecco’s modified Eagle medium (Invitrogen, Carlsbad, CA, USA). At 6, 12, 24, and 48 h postinfection (hpi), cells were harvested, and the RNA and DNA were extracted for detection of cytokine expression and viral load.

### Viral Load in SD0828-Infected Tissues and Cells

The viral DNA loads in the chicken and duck hearts, livers, spleens, lungs, kidneys, and brains as well as CEFs and DEFs and cloacal cotton swabs were extracted as described. Quantitative real-time PCR (qRT-PCR) primers of FAdV-4 were designed as described in a previous study (33) (Table 1), and it was performed using the ChamQTM SYBR^®^ qPCR Master Mix (Vazyme, Nanjing, China) and the 7500 Fast Real-Time PCR System (Applied Bio-systems, CA, USA). A 20-µL PCR tube was used under these specific conditions: (a) one cycle predenaturation at 95°C for 5 min, (b) 40 cycles denaturation at 95°C for 10 s, and (c) extension at 60°C for 34 s. The dissociation curve was then analyzed, processing each sample in triplicate.

### The Innate Immune Response of the SD0828-Infected Samples

Using qRT-PCR, mRNA expressions of the innate immune-related genes in livers, spleens, and PBMCs were ascertained. The primers for chicken β-actin, TLR3, TLR7, MDA5, IFN-α, IFN-β, IFN-γ, IL-1β, IL-6, IL-8, OAS, and Mx genes were partially referenced to the previous study (34), and the remaining were designed using Primer3 software (http://bioinfo.ut.ee/primer3-0.4.0/). The primers for duck β-actin, TLR3, TLR7, MDA5, RIG-I, IFN-α, IFN-β, IFN-γ, IL-1β, IL-6, IL-8, OAS, PKR, and Mx genes were referenced to the previous study (23, 35). The qRT-PCR method and reaction conditions were as described. All qRT-PCR primer information is shown in Table 1.

### The Antiviral Ability of Duck RIG-I in Infected CEFs

To further investigate the anti-FAdV-4 ability of duck RIG-I *in vitro*, CEFs in 6-well plates were transfected with the vectors PCAGGS, PCAGGS-RIG-I, and PCAGGS-RIG-I-CARD. At 24 h posttransfection, cells were infected with 10^3.3^ TCID_50_/mL SD0828 over 1 h of adsorption. At 6, 12, 24, and 48 hpi, cell samples were then collected for DNA extraction, which was performed as described. Viral titers were determined using qRT-PCR methods as described.

### Statistical Analysis

The relative mRNA expressions of the target genes in the infected and control groups were calculated based on the 2^−ΔΔCt^ method and quantified relative to β-actin, which was employed as the endogenous control to normalize expression levels of innate immune-related genes. The fold changes were logarithmically transformed. All data, expressed as means ± SDs, were analyzed using the Student’s *t*-test as determined by Graph Pad Prism 5.0 (Graph Pad Software Inc., San Diego, CA, USA). Statistical significance was recognized when *P* < 0.05 or *P* < 0.01.

## Results

### Phylogenetic and Genetic Analysis of the SD0828 Strains

**Figure 1:**
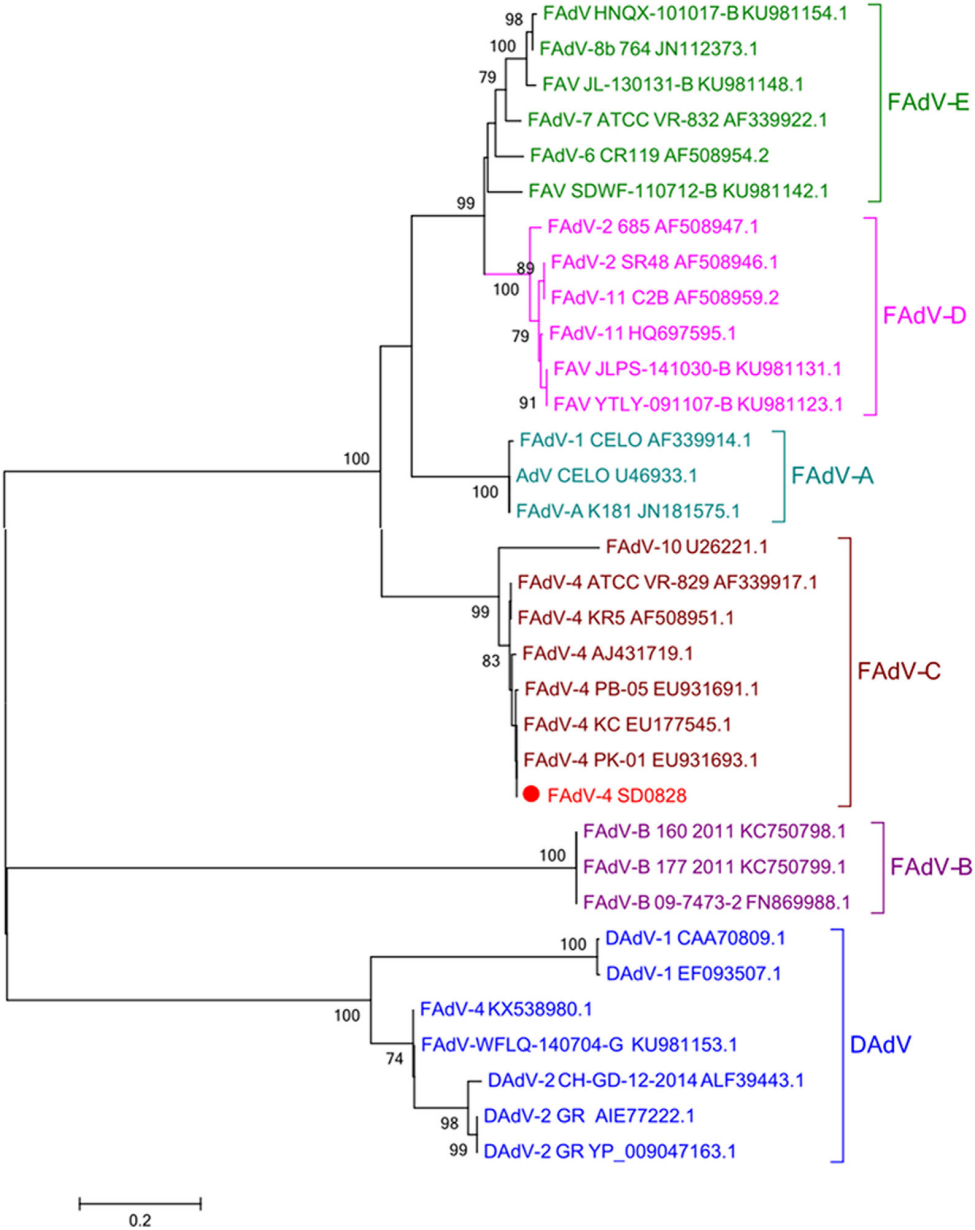
Phylogenetic tree of the isolated FAdV-4 strain (SD0828) based on the L1 loop of hexon gene sequences. Phylogenetic analysis was constructed based on the L1 loop of hexon DNA sequences of the reference strain CELO. The SD0828 sequenced in this study is marked with a solid red circle; other FAdV strains were found on the National Center for Biotechnology website and are referenced to previous studies. GenBank numbers are shown in Table 2. This phylogenetic tree was generated using MEGA 5.1 software employing the neighbor-joining method and a 1,000 bootstrap analysis. The scale bar is 0.2.

As the most abundant viral surface protein, the L1 loop of the hexon contains the major antigenic determinants, and in previous studies, AdV was also genotyped by building the phylogenetic tree of this region (8, 9). In this study, the entire hexon gene sequence of SD0828 consisted of 2,814 base pairs encoding 938 amino acids. The L1 loop is positioned between base pairs 145 and 1,041 in the hexon, and it encodes 299 amino acids. The entire hexon gene sequence for the SD0828 strain has been submitted to the NCBI under GenBank number MF804504. Phylogenetic tree analysis, based on the L1 loop of the hexon protein, indicates six major groups ranging from FAdV-A to -E and DAdV. The viral strain SD0828 isolated in this study was determined to be part of the FAdV-C group, and it is closely genetically related to the chicken-source FAdV-4 strains (Figure 1). In addition, all the duck-source AdVs (AdV-1, AdV-2, and AdV-4) are belong to the DAdV group (Figure 1).

### Clinical Signs and Gross Pathology

**Figure 2:**
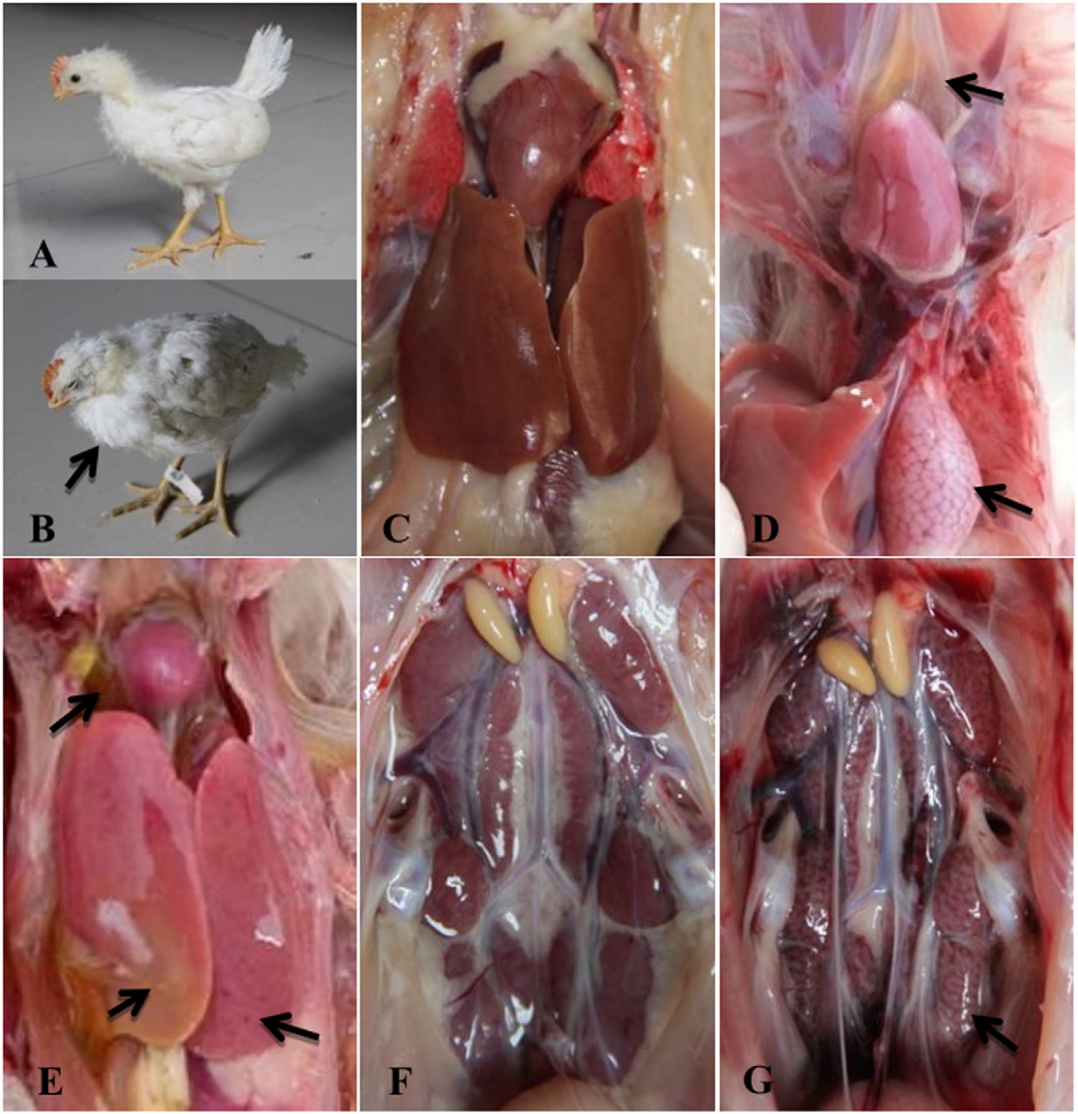
Clinical signs and gross lesions of FAdV-4 SD0828-infected chickens and uninfected controls. **(A)** Healthy chicken from the control group. **(B)** Depression and ruffling of neck feathers as seen in an SD0828-infected chicken. **(D)** Yellow jelly or watery-like exudate in the pericardial sac and glandular stomach swelling. **(E)** Swollen and pale liver. **(G)** Nephrotic kidney. **(C,F)** represent pericardia, livers, and kidneys of chickens from the control group.

At 1 dpi, all chickens from Group I exhibited clinical symptoms including depression, lethargy, ruffling of neck feathers, and decreased feed intake (Figure 2B). During necropsy, the pericardium had a light yellow jelly or water-like transparent exudate, and the liver was swollen and pale (Figures 2D,E). Glandular stomach swelling (Figure 2D) and nephrotic (Figure 2G) also occurred. All thirty chickens in Group I died within 2 dpi. In Group II, the infected chickens showed clinical symptoms starting at 3 dpi, and all animals in this group died within 5 dpi. The clinical symptoms and pathological changes seen in Group II were similar to those in Group I (data not shown). No clinical symptoms or pathological lesions were observed in the control group (Figures 2A,C,F).

Although SPF ducks underwent the same treatment (the same infectious doses and routes) as the SPF chickens, no clinical symptoms or gross lesions were found in either treatment group at any sampling point. In addition, ducks in the infected and control groups survived the entire study period (28 days) without clinical symptoms.

### Histopathological Analysis

**Figure 3:**
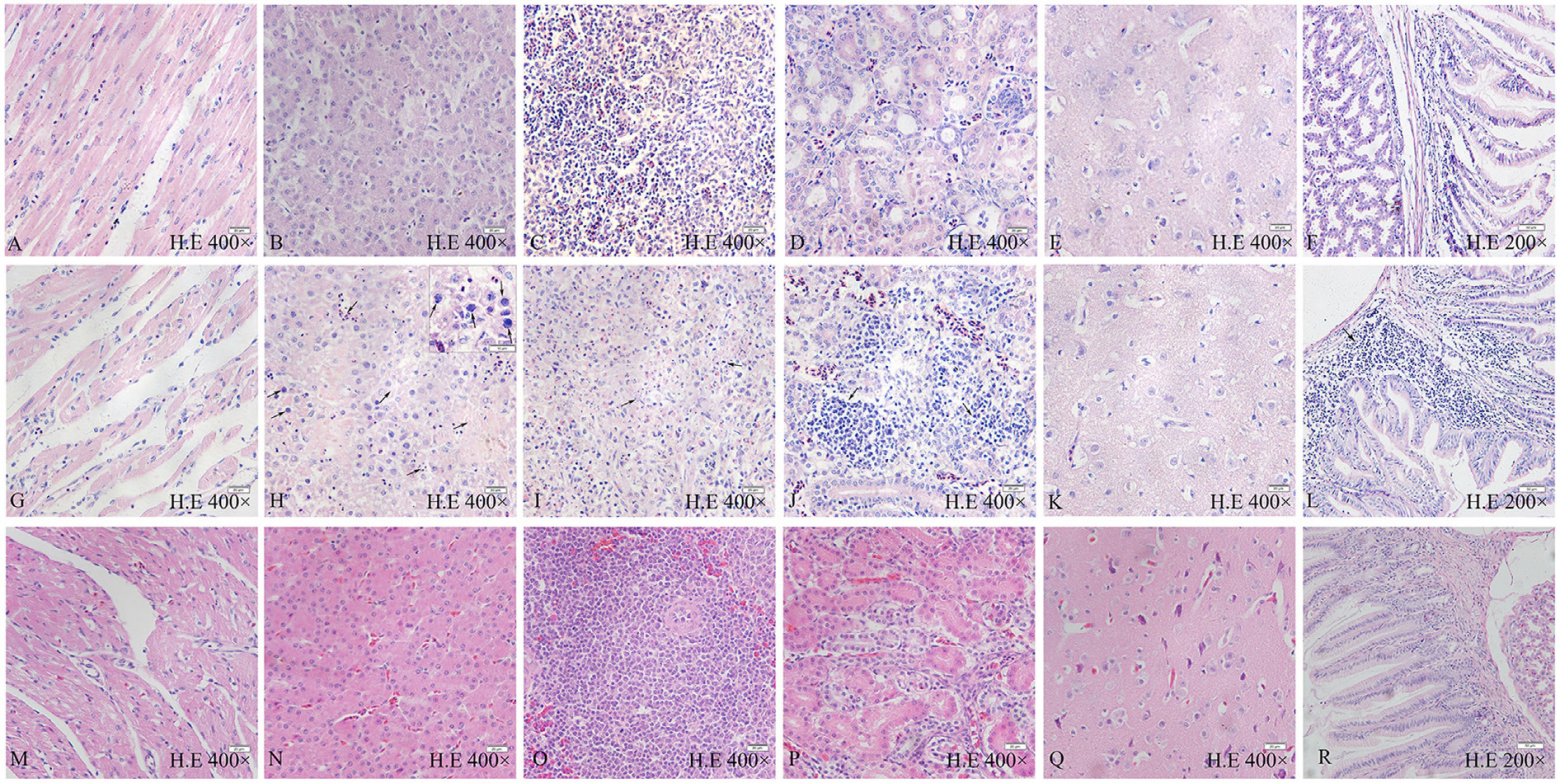
Pathological changes of FAdV-4 SD0828-infected chickens and ducks at different time points. **(A–F)** Heart, liver, spleen, kidney, brain, and glandular stomach, respectively, of chicken are from the control group. **(G–L)** Heart, liver, spleen, kidney, brain, and glandular stomach, respectively, are from the infected chicken. **(M–R)** Heart, liver, spleen, kidney, brain, and glandular stomach, respectively, are from the infected duck. **(G)** No obvious histologic lesions in the heart. **(H)** Large number of basophilic inclusion bodies and the vacuoles and hepatocyte necrosis (karyon pyknosis, karyorrhexis, and karyolysis) in the liver. **(I)** Massive loss of lymphocytes and obvious lymphocytic necrosis (karyon pyknosis, karyorrhexis, and karyolysis) in the spleen. **(J)** Severe lymphocytic infiltration in the kidney. **(K)** No obvious histological lesions in the brain. **(L)** Intense lymphocytic infiltration between the lamina propria and gland ducts in the glandular stomach. H&E stain, the original magnification of each organization is shown in the diagram, the scale bar = 50 µm in the **(F,L,R)** = 20 µm in the **(A–E,G–K,M–Q)** = 10 µm in the small box of **(H)**.

All chickens infected with SD0828 in Group I presented severe histopathological changes in various tissues compared to controls, as shown in Figures 3A–F, representing their hearts, livers, spleens, kidneys, brains, and glandular stomachs. No obvious histological heart lesions were found (Figure 3G). The most obvious change was a large number of basophilic inclusion bodies that were found in hepatocytes when observed *via* light microscopy under oil immersion (Figure 3H). Meanwhile, vacuolar degeneration and hepatocyte necrosis (karyon pyknosis, cataclasm, and karyolysis) occurred in the liver (Figure 3H). In the spleen, a massive loss of lymphocytes and obvious lymphocytic necrosis with karyon pyknosis, karyorrhexis, and karyolysis was seen (Figure 3I). Severe lymphocytic infiltration in the kidneys (Figure 3J) was observed as well. However, only slight lesions were observed in the brains of infected chickens (Figure 3K). In the glandular stomach, intense lymphocytic infiltration occurred between in the lamina propria and gland ducts (Figure 3L). In infected ducks, no obvious histological lesions could be found in any tissue (Figures 3M–R). This result is consistent with the clinical findings.

In summary, SD0828 can cause severe histopathological changes in various tissues in chickens. Although pericardial effusion was considerable, significant pathological changes were not found in the heart. Aside from the significant pathological changes described, a large number of basophilic inclusion bodies were observed in the liver, indicating the liver is the target organ of SD0828 in chickens.

### Viral DNA Load in the Infected Samples

**Figure 4:**
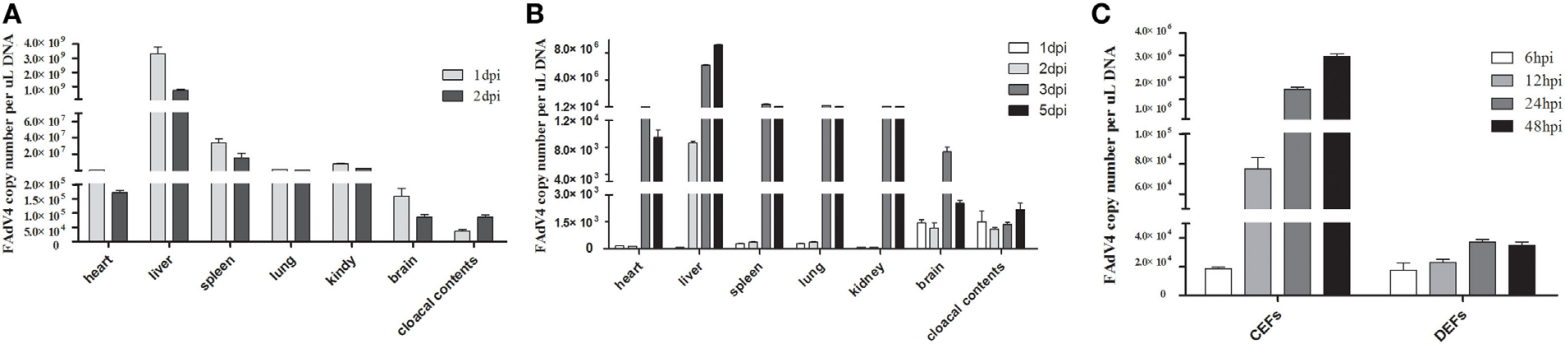
FAdV-4 SD0828 DNA loads in samples from infected fowl. **(A)** FAdV-4 SD0828 DNA loads in the tissues and cloacal cotton swabs from intramuscularly infected chickens. **(B)** FAdV-4 SD0828 DNA loads in the tissues and cloacal cotton swabs from orally infected chickens. Data are expressed as means ± SDs (*n* = 3). Three infected chickens were randomly selected for detecting viral DNA loads using real-time quantitative reverse transcription polymerase chain reaction (qRT-PCR) analysis. dpi, days postinfection. **(C)** FAdV-4 SD0828 DNA loads in infected chicken embryo fibroblasts (CEFs) and duck embryo fibroblasts (DEFs). Data are expressed as means ± SDs (*n* = 3), and infected CEFs and DEFs at 6, 12, 24, and 48 hpi were collected for detecting viral DNA loads using qRT-PCR analysis. hpi, hours postinfection.

As shown in Figure 4, the number of copies of this viral genome was determined in tissues, cloacal cotton swabs, and cells. In chicken Groups I and II, SD0828 quickly invaded and replicated in many tissues, including the target, immune, and central nervous organs. Because the liver contained the greatest viral load [3.31 × 10^9^ copies/μL in Group I at 1 dpi (Figure 4A) and 9.25 × 10^7^ copies/μL at 5 dpi in the Group II (Figure 4B)], it appeared to be the target organ. Although the inoculated virus stock was same in both groups, replication was relatively slow in Group II (oral) compared to Group I (IM; Figures 4A,B). In Group I, viral loads peaked at 1 dpi and generally declined at 2 dpi (Figure 4A). However, with the increase in virus clearance (cloacal contents) the tissue viral loads decreased significantly at 2 dpi (Figure 4A). In Group II, most tissues showed low viral loads until 3 dpi, when peaks were reached (Figure 4B). But in the liver, the SD0828 was could quickly replicated, and high loads were maintained from 2 to 5 dpi (Figure 4B). No viral DNA was detected in any tissues in the control group. In ducks, no virus was detected in any sample from any group at any sampling point.

To compare the pathogenicity of SD0828 in chickens versus ducks, a series of *in vitro* experiments were performed over time, revealing an increasing load in both CEFs and DEFs from 6 to 48 hpi (Figure 4C). However, the replication speed in CEFs was obviously faster compared to that in DEFs. Viral load increased from 1.86 × 10^4^ to 2.95 × 10^6^ copies/μL in CEFs, whereas it increased from 1.74 × 10^4^ to 3.49 × 10^4^ copies/μL in DEFs (Figure 4C).

### The Innate Immune Response of the Livers, Spleens, and PBMCs from Infected Chickens and Ducks

**Figure 5:**
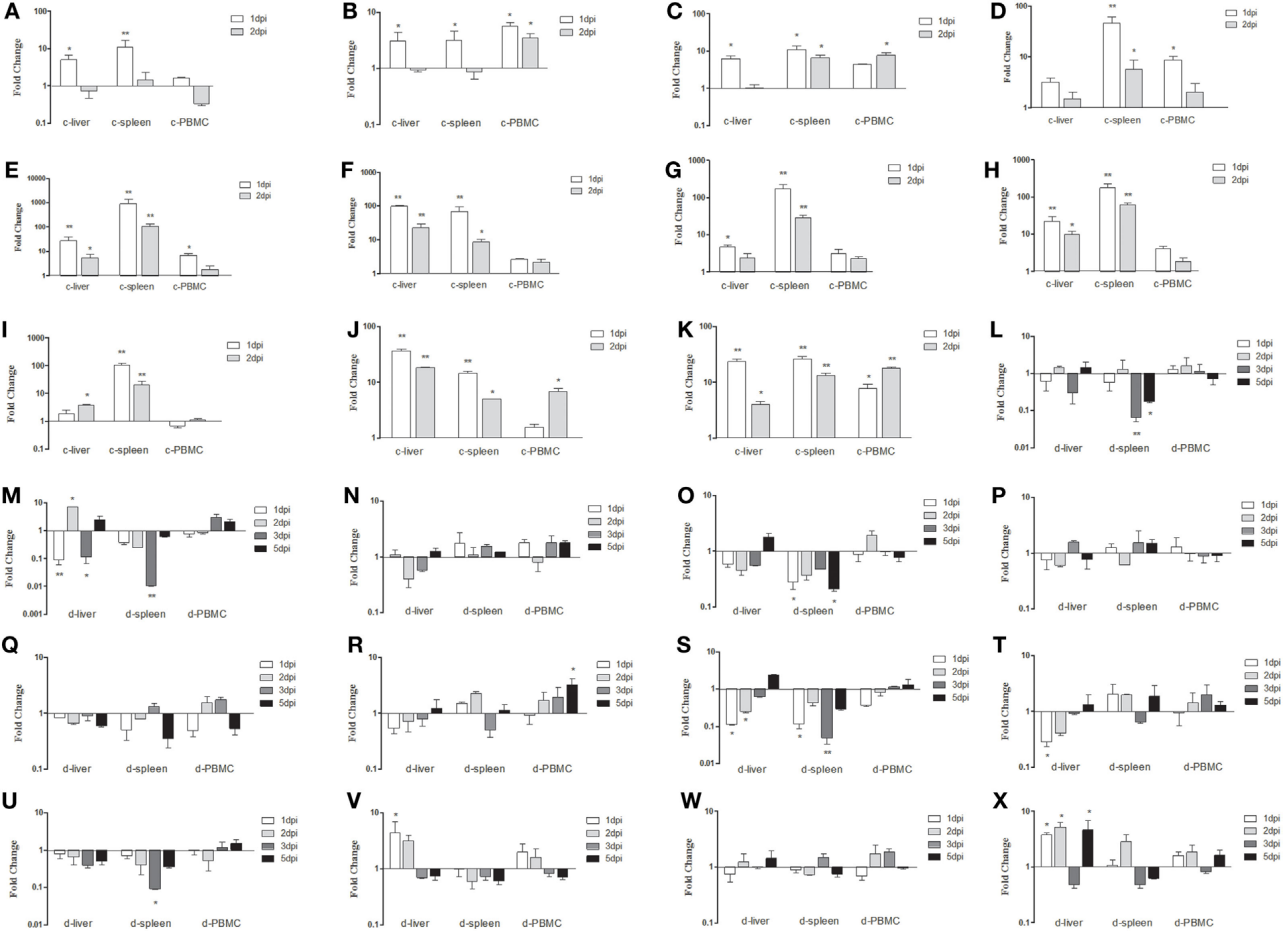
Expression profiles of immune-related genes in the livers, spleens, and peripheral blood mononuclear cells (PBMCs) from chickens and ducks infected intramuscularly. Tissue samples from chickens were collected at 1 and 2 dpi, and tissue samples from ducks were collected at 1, 2, 3, and 5 dpi. The inducible gene mRNA expression levels in the tissue samples were tested using real-time quantitative reverse transcription polymerase chain reaction (qRT-PCR) analysis. Relative expression levels were normalized to the β-actin gene and calculated using the 2^−ΔΔCt^ method. Data are expressed as means ± SDs (*n* = 3). **(A–K)** Expression levels of toll-like receptors TLR3 and TLR7, melanoma-differentiation-associated (MDA) 5, interleukin (IL)-1β, IL-6, IL-8, interferon (IFN)-α, IFN-β, IFN-γ, OASL, and Mx in the livers and spleens of infected chickens. **(L–X)** Expression levels of TLR3, TLR7, MDA5, RIG-I, IL-1β, IL-6, IL-8, IFN-α, IFN-β, IFN-γ, OAS, Mx, and protein kinase (PKR) in the livers and spleens of infected ducks. Differences were analyzed using the Student’s *t*-test. *P < 0.05; **P < 0.01; c, chicken; d, duck; dpi, days postinfection.

To identify which PRRs, cytokines, and IFN-stimulated genes (ISGs) could be involved in the antiviral innate immune response, mRNA expressions of TLR3, TLR7, MDA5, RIG-I, IL-1β, IL-6, IL-8, IFN-α, IFN-β, IFN-γ, Mx, PKR, and OAS (ducks)/OASL (chickens) in infected livers, spleens, and PBMCs were examined. In Group I chickens, mRNA expressions of TLR3 and TLR7 showed similar trends in the liver and spleen, both significantly increasing at 1 dpi and returning to the baseline levels at 2 dpi (Figures 5A,B). However, MDA5 in the spleen showed sustained upregulation from 1 to 2 dpi (Figure 5C). Also in the spleen, IL-1β mRNA expression showed significant (46.55-fold) upregulation, particularly at 1 dpi (*P* < 0.01; Figure 5D). The greatest increase occurred in IL-6, which showed a 909.51-fold increase at 1 dpi (*P* < 0.01; Figure 5E). Increases were also seen in IL-8 mRNA expression levels in the liver and spleen, which were comparable to one another, the liver showing the greatest upregulation (99.30-fold) at 1 dpi (*P* < 0.01; Figure 5F). The IFN transcript levels were significantly higher in the spleen than in the liver, and all IFN expressions were elevated in both organs (Figures 5G–I). In the spleen, mRNA expressions of IFN-α, IFN-β, and IFN-γ were upregulated 171.01-fold, 177.69-fold, and 104.17-fold, respectively at 1 dpi (*P* < 0.01; Figures 5G–I). Significant increases were also seen in OASL and Mx mRNA expressions at all sampling points in both organs (between 4.92-fold and 36.90-fold, *P* < 0.01; Figures 5J,K). In Group I ducks, mRNA expressions of TLR3, TLR7, MDA5, IL-1β, IL-6, and IL-8 remained at levels near those of controls in the liver and spleen. No significant differences between diseased and control ducks were found (*P* > 0.05, Figures 5L–N,P–R). However, mRNA expression levels of RIG-I were characterized by downward trends (Figure 5O). Different from the stable IFN upregulation in chickens, IFN expressions were primarily downregulated at varying degrees in the ducks (Figures 5S–U). Although most trends were not significant, several noticeable reductions in IFN-α and IFN-γ could be seen in the liver and spleen (Figures 5S,U). Expressions of both OAS and Mx were similar to those in controls across all sampling points in both the liver and spleen except in the liver at 1 and 2 dpi (Figures 5V,W). Finally, PKR mRNA expression was significantly upregulated at 1, 2, and 5 dpi in the liver when compared to controls (Figure 5X).

**Figure 6:**
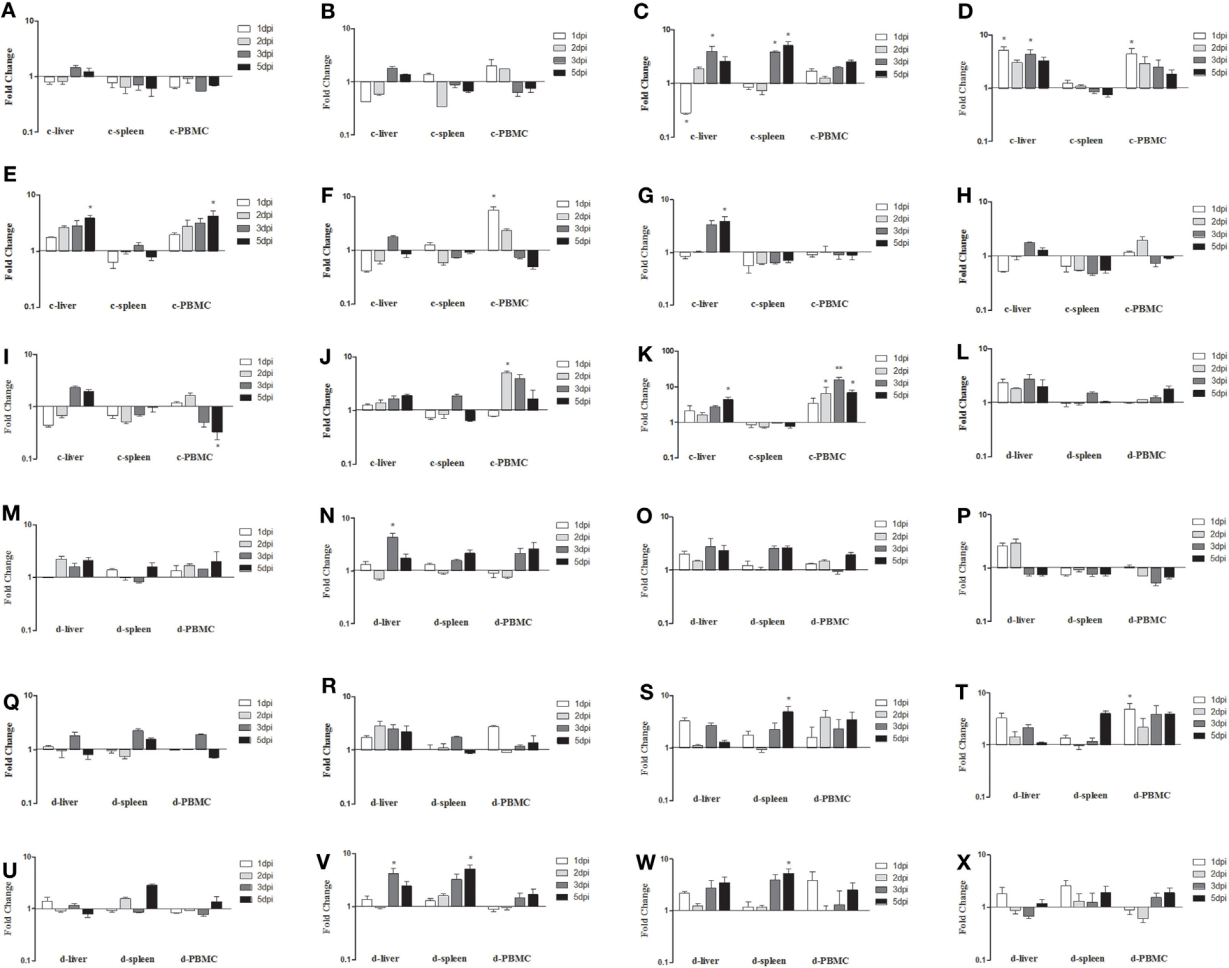
Expression profiles of immune-related genes in the livers, spleens, and peripheral blood mononuclear cells (PBMCs) from orally infected chickens and ducks. Tissue samples from chickens were collected at 1 and 2 dpi, and tissue samples from ducks were collected at 1, 2, 3, and 5 dpi. The inducible gene mRNA expression levels in tissue samples were tested using real-time quantitative reverse transcription polymerase chain reaction (qRT-PCR) analysis. Relative expression levels were normalized to the β-actin gene and calculated using the 2^−ΔΔCt^ method. Data are expressed as means ± SDs (*n* = 3). **(A–K)** Expression levels of toll-like receptors TLR3 and TLR7, melanoma-differentiation-associated (MDA) 5, interleukin (IL)-1β, IL-6, IL-8, interferon (IFN)-α, IFN-β, IFN-γ, OASL, and Mx in the livers and spleens of infected chickens. **(L–X)** Expression levels of TLR3, TLR7, MDA5, RIG-I, IL-1β, IL-6, IL-8, IFN-α, IFN-β, IFN-γ, OAS, Mx, and protein kinase (PKR) in the livers and spleens of infected ducks. Differences were analyzed using the Student’s *t*-test. *P < 0.05; **P < 0.01; c, chicken; d, duck; dpi, days postinfection.

As shown in Figure 6, the expressions of innate immune-related genes underwent relatively mild changes in samples from orally infected fowl. Among them, mRNA expressions of TLR3, TLR7, IFN-α, IFN-β, and IFN-γ in the livers, spleens, and PBMCs of infected chickens remained near the background level (*P* > 0.05, Figures 6A,B,G–I). Although the fold change trends in chickens infected *via* the IM and oral routes were similar for mRNA expressions of MDA5, IL-1β, IL-6, IL-8, OASL, and Mx, they were obviously small in the orally infected chickens (Figures 6C–F,J,K). Because no disease was seen in SD0828-infected ducks, changes in the tested innate immune-related genes in their livers, spleens, and PBMCs were small up to 5 dpi (*P* > 0.05, Figures 6L–X). These results indicate that chickens infected with SD0828 significantly upregulate mRNA expression levels of IL-6 IL-8, IFNs, Mx, and OASL; however, ducks infected with the same do not, showing only small changes in these cytokines and ISGs.

### The Innate Immune Responses of Infected CEFs and DEFs

**Figure 7:**
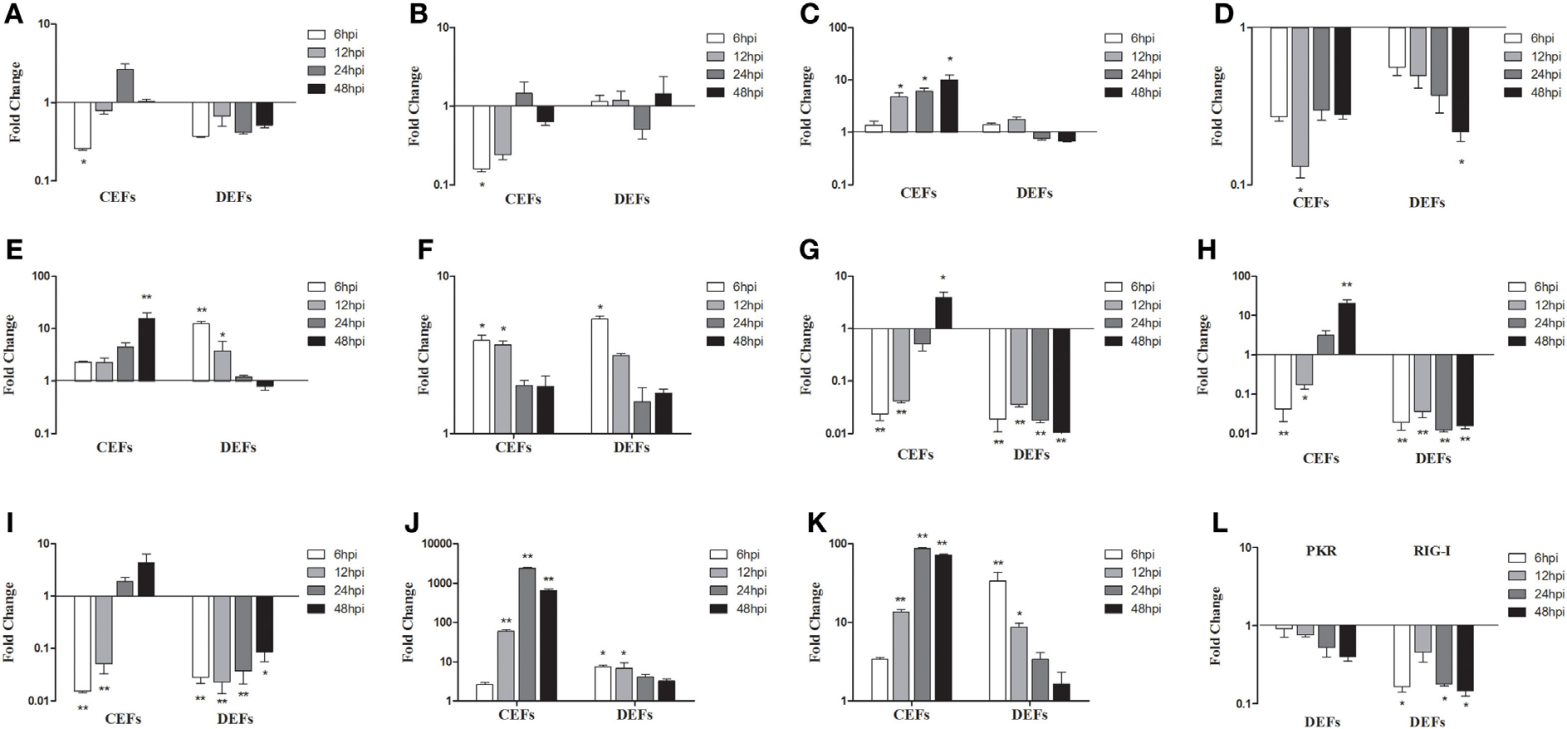
Expression levels of immune-related genes in infected chicken embryo fibroblasts (CEFs) and duck embryo fibroblasts (DEFs). Cells were infected with FAdV-4 SD0828 and then collected at 6, 12, 24, and 48 hpi. The inducible gene expression levels in the cells were tested using real-time quantitative reverse transcription polymerase chain reaction analysis. Fold change was calculated by comparing the experimental group with controls at the same sampling point, and relative expression levels were normalized to the β-actin gene and calculated using the 2^−ΔΔCt^ method. Data are the means across three independent experiments, and each experiment was analyzed in triplicate. Results are expressed as means ± SDs (*n* = 3). The Student *t* test was performed to evaluate differences. **(A–K)** Expression levels of TLR3, TLR7, MDA5, IL-1β, IL-6, IL-8 IFN-α, IFN-β, IFN-γ, Mx, and OASL in CEFs and DEFs. **(L)** Expression levels of PKR and RIG-I in DEFs. *P < 0.05; **P < 0.01; hpi, hours postinfection.

To further study SD0828-induced innate immune responses, CEFs and DEFs were infected. At 6, 12, 24, and 48 hpi, cells were collected and examined for gene expression. In CEFs, mRNA expressions of TLR3 and TLR7 were significantly downregulated at 6 hpi after which, changes were not significant (Figures 7A,B). Conversely, the expression of MDA5 was significantly enhanced at 12 and 48 hpi (Figure 7C). Although the proinflammatory cytokine IL-1β was downregulated, IL-6 and IL-8 were upregulated over the entire sampling period (Figures 7D–F). The three IFNs were downregulated in the early stages and upregulated at 24 and 48 hpi (Figures 7G–I). Furthermore, Mx and OASL were highly expressed (Figures 7J,K).

In DEFs, mRNA expressions of TLR3, TLR7, and MDA5 were much as seen in infected duck livers and spleens (Figures 7A–C), but IL-6, IL-8, Mx, and OAS were expressed differently (Figures 7E,F,J,K). Unlike the *in vivo* results, IL-6 was upregulated at 6 and 12 hpi (Figure 7E) as was IL-8 at 6 hpi (Figure 7F) and Mx and OAS at 6 and 12 hpi (Figures 7J,K). Furthermore, mRNA expressions of IL-6, IL-8, Mx, and OAS exhibited changes in a time-dependent manner: the fold increase gradually decreased with time (Figures 7E,F,J,K). However, IFN expressions showed more pronounced downregulation compared to *in vivo* results (Figures 7G–I). In addition, the PKR and RIG-I also show different degrees of down-regulation (Figure 7L).

### Antiviral Activity of Duck RIG-I and Its CARD Domain in Infected CEFs

**Figure 8:**
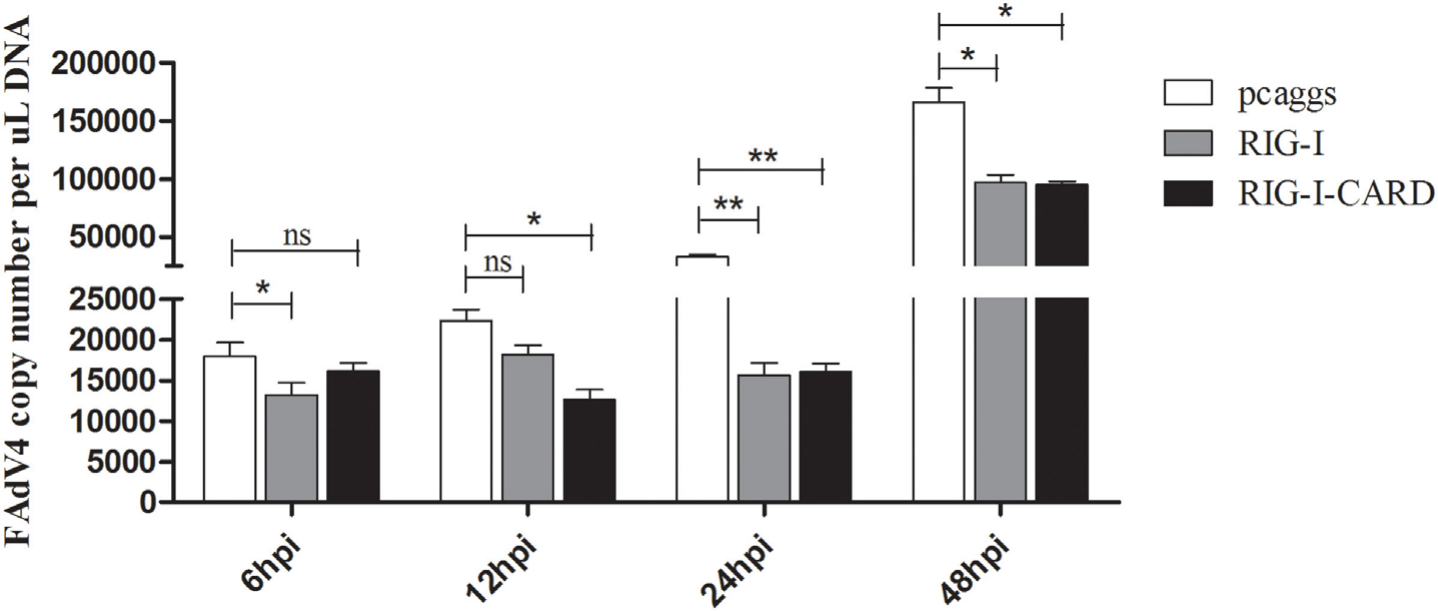
Antiviral assays of duck retinoic acid-inducible gene I (RIG-I)-mediated in chicken embryo fibroblasts (CEFs). The empty vector PCAGGS, PCAGGS-RIG-I, and PCAGGS-RIG-I-CARD were transfected into CEFs. At 24 h posttransfection, cells were infected with 500 μL/well virus diluent at 10^3.3^ TCID_50_/mL. Cells were collected for detecting viral titers at 6, 12, 24, and 48 hpi using real-time quantitative reverse transcription polymerase chain reaction analysis repeated three times, and each sampling point was analyzed in triplicate. Results are expressed as means ± SDs (*n* = 3). The Student’s *t*-test was performed to evaluate differences. *P < 0.05; **P < 0.01; hpi, hours postinfection.

Previous reports suggest that RIG-I exist in ducks but not in chickens (25); therefore, the antiviral capabilities of duck RIG-I and its CARD domain were examined in CEFs. Results show that SD0828 titers in duck RIG-I and RIG-I-CARD-transfected CEFs were significantly lower than in empty vector-transfected cells (Figure 8). Furthermore, the antiviral effects of duck RIG-I and its CARD domain were most pronounced at 24 hpi (Figure 8). Our data suggest that duck RIG-I and RIG-I-CARD can significantly inhibit viral replication, possibly explaining why SD0828 viral titers are much higher in CEFs than in DEFs.

## Discussion

Since 2014, HHS caused by FAdV-4 has undergone a pandemic outbreak in China and has caused high mortality in broiler chickens, thus causing huge losses for the Chinese poultry industry (7). In some studies, chickens were infected with FAdV-4 *via* IM, oral, or intranasal routes (17, 36–38). When compared with the oral and intranasal routes, IM produces stronger pathogenicity (17, 37). In duck AdV infection models, the IM infection route was often used (27). Therefore, to better understand the various pathogenicity of FAdV-4 in SPF chickens and ducks, we chose the IM and oral routes for infection.

In this study, infected SPF chickens exhibited typically in both clinical signs and gross and histopathological lesions; however, no clinical signs or histopathological lesions were found in infected SPF ducks when exposed at the same doses compared to chickens. Considering that the age at infection for ducks might have been too great, we infected 3- and 7-day-old ducklings with the dosage used with adult ducks, but no disease or clinical illness was observed, and no virus was detected (data not shown). One possible reason could be the low infection dose; therefore, 3-week-old ducks were infected with a dose 20 times that used with the chickens, but signs of disease and clinical illness remained absent (data not shown). These results are consistent with those found in a previous study (39). However, in Chen’s study, the duck source FAdV-4-infected ducks showed typical hydropericardium and death with the intracerebral infection route used (28). Why does the same viral cause such different reactions in chickens and ducks? Species diversity in addition to differences in viral receptors between chickens and ducks could be the answer. Additionally, the viral strain used in this study was isolated from clinically diseased chickens, presenting another possible answer.

In infected chickens, SD0828 could quickly replicate in many tissues. Among them, the liver showed the highest viral load followed by the spleen. Viral loads in various tissues were consistent with those found in a previous study (33, 39). Also consistent with a previous study, a large number of basophilic inclusion bodies in the liver were observed (38). Although a large amount of effusion was seem in the pericardium, no significant pathological changes were found in the heart. Pan et al. reported that although no clinical signs were observed in infected ducks, but viral replication was detected (39). However, we found no viral replication in the tissues or cloacal contents of infected ducks. We speculate that this difference could be the result of the differing infection routes between the two studies. In addition, even though SD0828 can replicate in both CEFs and DEFs, the rate of replication was significantly slower in DEFs compared to CEFs. These results indicate that CEFs are more amenable to efficient viral growth.

By comparing the results of *in vivo* and *in vitro* experiments, we gain a better understanding of SD0828’s pathogenicity. As our results show, the expression trends of PRRs, cytokines, and ISGs are similar between the tissues from IM-infected SPF chickens and CEFs. The most obvious changes were the highly significant upregulations of IL-6, IL-8, Mx, and OASL. As seen with the major proinflammatory cytokines, the highly significant upregulated expressions of IL-6 and IL-8 induced severe inflammatory responses in infected tissues, eventually leading to death at a rapid rate. This is consistent with responses induced by avian influenza and duck Tembusu viral infections (35, 40, 41). Although ISG mRNA expression levels also showed significant upregulation, they did not withstand the “inflammatory storm” in infected organisms. This finding is also consistent with Chan’s study in which inducing Mx and PKR failed to protect chickens from highly pathogenic H5N1 AIV infection (40). The PBMCs, as the innate immune cells in the circulatory system, express innate immune-related genes. Changes in these expressions have important implications for an innate immune response caused by the virus. Results show that trends in the expressions of innate immune-related genes in PBMCs are similar to those seen in the liver and spleen. They also show that the innate immune responses induced in orally infected chickens are significantly lower than those induced in the IM-infected chickens.

It is noteworthy that the innate immune response in infected SPF ducks and DEFs was different. In ducks, the mRNA expressions of PRRs, cytokines, and ISGs showed minimal changes; however, in infected DEFs, IL-6, IL-8, Mx, and OAS mRNA expressions were significantly upregulated. Even when infected *via* the IM route, viral replication was not detected in duck livers or spleens. Thus, the innate immune responses in these two tissues were not triggered. While SD0828 could proliferate in DEFs, the innate immune response was triggered by the process of viral entry into cells and subsequent proliferation. Although the mRNA expression levels of IL-6, IL-8, Mx, and OAS were shown to be upregulated in both CEFs and DEFs, their tendencies differed. The fold increases in IL-6, Mx, and OAS rose with time in CEFs, but they did not rise as high in DEFs. These results suggest that the immune response induced by the same virus at the same dose differs according to cell type. It is generally believed that many ISGs were induced by IFN (42, 43), but we found that the expression levels of Mx and OSA were significantly upregulated while those of IFNs were downregulated in DEFs. We conclude that pathways other than IFN-associated pathways could induce ISG expression.

Although RIG-I primarily consisted of viral dsRNAs and 5′-triphosphate groups in the cytoplasm (24), our previous studies have shown that both RIG-I and RIG-I-CARD have significant antiviral roles postinfection in DNA-viral-(duck plague virus)-infected DEFs (23). Similarly, these experiments show that duck RIG-I and RIG-I-CARD have significant antiviral effects in SD0828-infected CEFs. This could be why the replication capability of SD0828 is greater in CEFs than in DEFs.

In summary, FAdV-4 SD0828 can cause HHS, and this can lead to massive and rapid death among infected SPF chickens. In contrast, infected SPF ducks barely exhibit clinical signs or gross and histopathological lesions, and the virus cannot be detected in their organs. By detecting the viral DNA loads and innate immune-related gene expression levels in infected tissues, we speculate that the rapid replication of these viruses in chickens and the excessive expression of inflammatory cytokines (IL-6 and IL-8) could trigger death. This could explain why the viral titer in CEFs is greater than that in DEFs.

## Ethics Statement

All animals used in this study were handled in strict accordance with the guidelines of Shandong Agricultural University Animal Care and Use Committee. The approval number was SDAU-2016-001.

## Author Contributions

RL and GL performed most of the experiments, wrote the manuscript, and made pathological sections. JL and SH collected samples and extracted the tissues RNA and DNA. XH and HW kept animals. MG performed the data calculation with support from ZL and NL. YS reviewed and polished the article. TC and LW designed the study.

## Conflict of Interest Statement

The authors declare that the research was conducted in the absence of any commercial or financial relationships that could be construed as a potential conflict of interest.
